# EARLY PALLIATIVE CARE UTILIZATION AMONG OLDER ADULTS WITH METASTATIC CANCER IN A MEDICARE COHORT

**DOI:** 10.1093/geroni/igae098.2495

**Published:** 2024-12-31

**Authors:** Molly Nowels, Elissa Kozlov, Stephen Crystal, Paul Duberstein

**Affiliations:** Weill Cornell Medicine, United States; Rutgers, The State University of New Jersey, Piscataway, New Jersey, United States; Rutgers, The State University of New Jersey, New Brunswick, New Jersey, United States; Rutgers, The State University of New Jersey, Piscataway, New Jersey, United States

## Abstract

Meta-analytic data have found early palliative care (EPC) to be associated with better outcomes among older adults with cancers. EPC is now recommended for all people with advanced cancers. Ideally, the resources required to appropriately treat referred patients would reflect the needs of the patient population. We are aware of no prior attempts to characterize patients receiving EPC. To address this gap, we used a 20% random sample of fee-for-service Medicare enrollees from 2008-2016 to identify predictors of EPC use within 8 weeks of a new diagnosis of metastatic cancer. Logistic regression modeling adjusted for demographics, cancer site, indicators for a variety of physical and behavioral health diagnoses, the Elixhauser comorbidity index, and several indicators for past-year health service use. 525,936 individuals were included, with 4.9% (25,982) receiving EPC. Several behavioral health diagnoses were associated with increased odds of EPC, including both active and remitted alcohol use disorder; active drug use disorder; major depression; and schizophrenia. These findings suggest that the caseloads of palliative care clinicians may disproportionately include persons with histories of significant psychiatric disorders and behavioral health disorders. Though palliative care clinicians may effectively manage some psychological symptoms, such interventions fail to consistently do so. Moreover, few palliative care teams include psychologists or psychiatrists, potentially leading to significant gaps in clinical expertise. Models of palliative care should integrate specialty mental health clinicians to meet the demand for EPC services and manage the spectrum of needs among older adults with advanced cancers.

